# Atrioventricular node ablation for atrial fibrillation in the era of conduction system pacing

**DOI:** 10.1093/eurheartj/ehae656

**Published:** 2024-10-14

**Authors:** Jacqueline Joza, Haran Burri, Jason G Andrade, Dominik Linz, Kenneth A Ellenbogen, Kevin Vernooy

**Affiliations:** Department of Medicine, McGill University Health Center, Montreal, Quebec, Canada; Cardiology Department, University Hospital of Geneva, Geneva, Switzerland; Department of Medicine, Vancouver General Hospital, Vancouver, British Columbia, Canada; Department of Cardiology, Cardiovascular Research Institute Maastricht (CARIM), Maastricht University Medical Center, Maastricht, The Netherlands; Virginia Commonwealth University Medical Center, Richmond, VA, USA; Department of Cardiology, Cardiovascular Research Institute Maastricht (CARIM), Maastricht University Medical Center, Maastricht, The Netherlands

**Keywords:** Atrial fibrillation, Catheter ablation, Conduction system pacing, Atrioventricular node ablation

## Abstract

Despite key advances in catheter-based treatments, the management of persistent atrial fibrillation (AF) remains a therapeutic challenge in a significant subset of patients. While success rates have improved with repeat AF ablation procedures and the concurrent use of antiarrhythmic drugs, the likelihood of maintaining sinus rhythm during long-term follow-up is still limited. Atrioventricular node ablation (AVNA) has returned as a valuable treatment option given the recent developments in cardiac pacing. With the advent of conduction system pacing, AVNA has seen a revival where pacing-induced cardiomyopathy after AVNA is felt to be overcome. This review will discuss the role of permanent pacemaker implantation and AVNA for AF management in this new era of conduction system pacing. Specifically, this review will discuss the haemodynamic consequences of AF and the mechanisms through which ‘pace-and-ablate therapy’ enhances outcomes, analyse historical and more recent literature across various pacing methods, and work to identify patient groups that may benefit from earlier implementation of this approach.

## Graphical Abstract

**Graphical Abstract:**
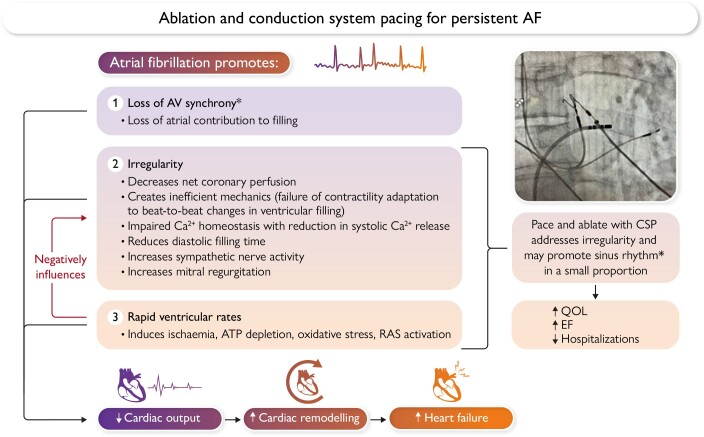
The mechanisms through which the pace-and-ablate strategy influences the hemodynamic effects of atrial fibrillation.

## Introduction

Atrial fibrillation (AF) is a common cardiac arrhythmia, resulting in substantial impairments in morbidity and mortality.^1^ While the management of paroxysmal AF has become increasingly straightforward, persistent AF continues to present a clinical dilemma, having undergone several substantial philosophical shifts in management over the last decade. Historically, the management of persistent AF was centred on pharmacological rate control strategies, with permanent pacemaker implantation and atrioventricular node ablation (AVNA) being considered a reasonable second-line strategy when drug therapy was ineffective or poorly tolerated. In these patients, AVNA was demonstrated to be highly effective in improving quality of life (QOL)^2,3^ while decreasing healthcare costs through a reduction in hospitalizations, outpatient visits, and antiarrhythmic drug use (AAD).^4^ However, concern regarding an increased risk of heart failure (HF) secondary to chronic right ventricular apical pacing-induced ventricular dyssynchrony limits the widespread use of this strategy. With the advent of conduction system pacing (CSP) targeting the His bundle or left bundle branch area, AVNA has seen a revival where ventricular dyssynchrony may be less of an issue.

This review will discuss the role of permanent pacemaker implantation and AVNA (‘pace-and-ablate’) for AF management in this new era of CSP. The haemodynamic consequences of AF and mechanisms by which the pace-and-ablate approach proposes to improve outcomes will be identified. Finally, a thorough analysis of the historical and more current literature on pace-and-ablate therapy across various pacing methods and the critical areas for research necessary to move this field forward will be considered.

## Persistent atrial fibrillation as a clinical problem

Atrial fibrillation ablation is an established rhythm control strategy to prevent recurrence. It has shown to be superior to AAD therapy in maintaining sinus rhythm and for symptomatic improvement when performed as either an initial (‘first-line’) or ‘second-line’ therapy (i.e. when antiarrhythmic drugs have been ineffective, are contraindicated, or produce intolerable adverse effects).^1,5^ However, left atrial catheter ablation is not universally curative. Several factors predictive of AF recurrence have been identified, including increasing age, female sex, increased left atrial dimensions, renal dysfunction, longer duration of AF, and the presence of coronary artery disease.^6^ In those with persistent AF, there is an average 40%–45% chance of maintaining sinus rhythm at two years following a single ablation procedure without the use of AADs.^7^ While success is improved with repeat ablation procedures and the concurrent use of AADs, the likelihood of maintaining sinus rhythm at 5 years is only 50% in those with persistent AF and decreases to 40% in those with long-standing persistent AF.^8^ The CABANA study has shown lower efficacy of catheter ablation in the elderly.^9^ Importantly, trials investigating patients with persistent AF ablation have been constrained by limited follow-up durations, with many patients still necessitating AAD therapy despite intervention. This potentially leaves many patients with symptomatic persistent AF without adequate relief where it may be reasonable to consider an earlier rate control approach with a pace-and-ablate strategy.^10^

## Haemodynamic consequences of atrial fibrillation: irregulopathy

Through excessive heart rates, beat-to-beat irregularity, absence of atrial contraction, and reduced coronary flow reserve, AF causes harmful effects upon left ventricular (LV) mechanical stretch and sympathetic nerve activity, leading to unfavourable haemodynamic consequences (*Graphical Abstract*). The cycle ensues with increased filling pressures and neurohormonal changes, resulting in atrial fibrosis and altered calcium handling, leading to further adverse cardiac remodelling, referred to as arrhythmia-induced cardiomyopathy.^11^

Early pioneering work attempting to elucidate the effect of ‘irregularity’ on intracardiac pressures and cardiac output (CO) began in 1983 when Naito *et al*.^12^ studied the haemodynamic effects of atrioventricular (AV) block in canine hearts. After open surgical AVNA was performed in 20 mongrels, ventricular pacing with a regular and irregular (five extra-stimuli introduced at varying coupling intervals) pattern was performed with each sequence from the left atrium and the LV apex: AV sequential pacing at 100 ms; ventricular-only pacing during sinus rhythm (with AV dissociation); AF with ventricular pacing; and AV sequential pacing but with an AV interval of −100 ms. They found that a normal AV sequence was of ultimate importance as AV sequential pacing resulted in optimal haemodynamics during both regular and irregular pacing. The most deleterious haemodynamic effects (reduced CO, increased left atrial pressure, and reduced LV pressure) were observed when atrial systole was superimposed on a ventricular contraction resulting in active retrograde atrial emptying, and although AV dissociation resulted in overall lower CO than during sequential AV pacing, only *irregularly* pacing the ventricle during AF resulted in a further drop in CO. The CO during this particularly deleterious sequence was indistinguishable from AF with irregular ventricular pacing. The loss of the atrial kick appeared to be the causal factor for the initial 22% reduction in CO, but pacing *irregularly* resulted in a further 9% CO drop. In other words, regular paced rhythms were still better than irregularly paced rhythms. Mitral valve regurgitation during angiography was observed in this study only in the presence of irregularity.

Further exploration into ‘irregulopathy’ was pursued by haemodynamic evaluation at the time of AVNA.^13,14^ Both regular and irregular ventricular pacing protocols at faster and slower heart rates were tested in patients. An irregular ventricular rhythm, *independent of rate*, was found to decrease the CO by 12%–15% when compared with a regular ventricular-paced rhythm.^13,14^ Irregular pacing also increased pulmonary capillary wedge pressure and right atrial pressure.^14^ Failure of cardiac contractility adaptation during beat-to-beat changes in ventricular filling may explain part of the mechanism for haemodynamic deterioration during irregular rhythms. The reduction in stroke volume that occurs with short RR intervals may not be entirely compensated for by the increase in stroke volume accompanying long RR intervals. Combined computational modelling of beat-to-beat speckle tracking on echocardiographic images confirms these findings, where beat-to-beat changes in preload explain the differences in LV systolic function, and that a reduced diastolic filling time can explain the variability of LV function in patients with AF.^15^ Geelen *et al*.^16^ further demonstrated consistent haemodynamic improvements over time post-AVNA,^16^ accompanied by improvements in cardiac index, and reductions in LV end-diastolic pressures and dimensions at 6 months.

Other underlying mechanisms for the reduced output attributed to irregularity invoke neurohormonal and vasomotor factors. A greater increase in atrial natriuretic peptide (ANP) is measured during irregular vs. regular ventricular pacing causing arterial and venous dilation with vagally mediated inhibition of cardiac sympathetic input. Plasma ANP and brain natriuretic peptide levels have been shown to progressively decrease after rate regularization post-AVNA.^17^

Irregularity may also reduce CO through a reduction in myocardial perfusion. Atrial fibrillation causes an increase in coronary flow that is *insufficient* to meet the increased myocardial oxygen demand. Using Doppler guide wires positioned in the left coronary system, coronary flow measurements were taken during sinus rhythm, induced AF, and right atrial pacing.^18^ The increase in coronary flow following AF induction is independent of the changes in heart rate and blood pressure, suggesting this increase is not proportional to that required by an augmented myocardial oxygen demand. Importantly, the loss of atrial contraction during AF induction did not affect the coronary vascular resistance, suggesting that the irregularity itself is solely responsible for the coronary vasoconstriction that acts in opposition to dilation during AF, thus impeding coronary flow and reducing coronary flow reserve.

An irregular rhythm also causes distinct functional and molecular LV remodelling in the absence of tachycardia. Irregularity is associated with alterations in ventricular cardiomyocyte Ca^2+^ haemostasis with a reduction in systolic Ca^2+^ release.^19^ A diminished Ca^2+^ load within the sarcoplasmic reticulum (SR) together with increased SR Ca^2+^ leak via hyperphosphorylation of the ryanodine receptor has been shown to lower the systolic Ca^2+^ amplitude. This reduction is a key participant in contractile dysfunction present in patients with HF.^20^ An increase in cytosolic Na^+^ causing action potential prolongation and oxidative stress within the LV myocardium further contributes to the Ca^2+^ handling changes in the AF ventricle.^19,21^

Increased sympathetic nerve activity is observed after AF induction which is in part attributable to the irregular ventricular response.^22^ Efferent post-ganglionic muscle sympathetic nerve activity was recorded from the left peroneal nerve in eight patients undergoing electrophysiology study. Patients underwent induction of AF, and in those who converted to sinus rhythm afterwards, irregular and regular RA pacing was performed. During AF, an increase in sympathetic activity was noted when compared with sinus rhythm. A further increase was noted during irregular pacing when compared with regular pacing without a significant change in blood pressure or central venous pressure. Given that increased sympathetic activity is known to be detrimental, particularly in patients with LV dysfunction, removing irregularity by restoration of sinus rhythm or through AV node ablation should help patients.

## Impacts of pace-and-ablate therapy on atrial fibrillation management

**Figure 1:**
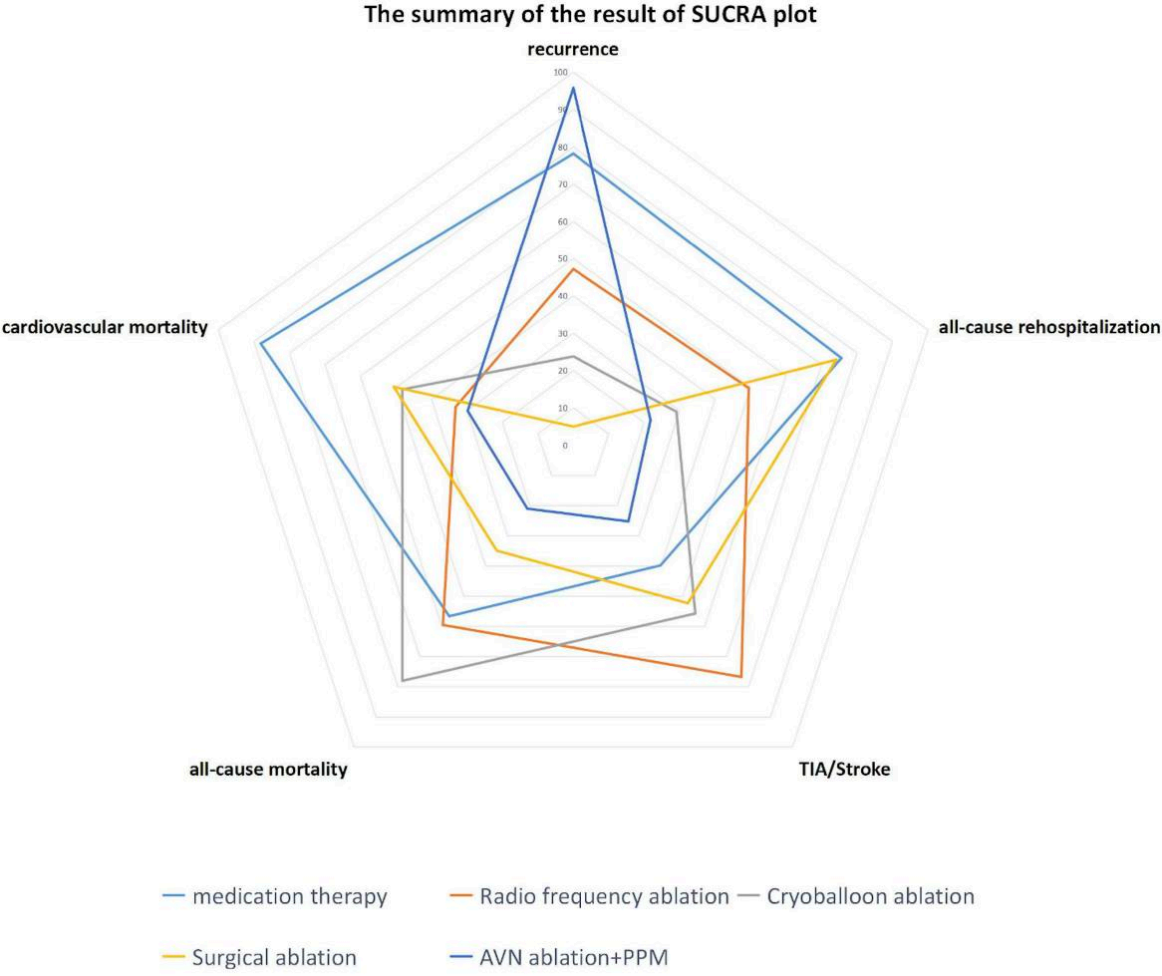
Radar plot indicating the overall risk of efficacy and safety endpoints from the different atrial fibrillation management strategies. It illustrates that with the exception of recurrences, pace-and-ablate strategy for atrial fibrillation has a good efficacy and safety profile. See permission letter from Professor Zeyi Cheng for *Figure 1*.^23^

A recent network meta-analysis aimed to compare various AF therapies, ranking the efficacy and safety among drug therapy, AF ablation strategies (RF, cryoballoon, and surgical ablation), and pace-and-ablate therapy. In a pairwise comparison, it was shown that although AF ablation strategies performed best in terms of reducing AF recurrence, a pace-and-ablate approach was consistently better than any other treatment in reducing cardiovascular and all-cause mortality, re-hospitalization, and stroke as shown in *Figure 1*.^23^

Interestingly, a recent prospective observational study of patients with persistent AF demonstrated spontaneous return to sinus rhythm in a small subset of patients after pace-and-ablate therapy with CSP.^24^ We speculate that the treatment of the irregulopathy favours normalization of atrial pressures and volumes, with a reduction in atrial stretch, which may lead to reverse atrial remodelling responsible for spontaneous restoration of sinus rhythm. Alternatively, the return to sinus rhythm may also simply reflect the routine chance of spontaneous conversion to sinus rhythm.

## Pace-and-ablate therapy in heart failure

A decision on rate or rhythm control with drugs or invasive treatment is complex and highlights the importance of an electrophysiologist in the multidisciplinary team setting to improve the decision-making process on arrhythmia management in patients with HF. There is mounting evidence that AF ablation improves clinical outcomes in paroxysmal and patients with persistent AF with HF.^25,26^ The patients included in these trials were relatively young without multiple comorbidities not necessarily reflecting the make-up of a typical HF clinic.^25^ The PABA-CHF trial showed superiority of pulmonary vein isolation (PVI) when compared with AVNA with biventricular cardiac resynchronization therapy (BIV-CRT) in patients with low ejection fraction (EF) in terms of improved EF, QOL, and 6-min walk scores, underscoring the importance of sinus rhythm when attainable.^27^ The average age of these patients was 60 years with left atrial diameters of 4.7 cm reflecting a population more likely to maintain sinus rhythm. Conversely, the RAFT-AF trial failed to demonstrate superiority of ablation-based rhythm management over rate control for the primary endpoint of HF hospitalization and mortality, although the trial was underpowered having stopped early due to recruitment concerns.^28^ Both studies had limited follow-up and the longer-term costs of re-hospitalization and repeat procedures were not considered.

The APAF-CRT trial highlighted the potential re-emergence of pace-and-ablate therapy in patients with HF.^29^ Atrioventricular node ablation and BiV-CRT implantation in patients with HF with permanent AF and at least one recent hospitalization were shown to reduce mortality compared with medical rate control over a follow-up of 4 years. It is worth noting that many patients included in this study had good rate control with medical therapy. The clinical benefit was hypothesized to result from the combination of rate lowering and rate regularization by AVNA in combination with BiV pacing to avoid ventricular dyssynchrony.

## Pacing strategy with atrioventricular node ablation

### Atrioventricular node ablation and right ventricular pacing

For decades, the right ventricle (RV) has been the preferred location for pacing given its reliability, stability of lead parameters over time, and easy accessibility. Early studies evaluating the role of AVNA and RV pacing (RVP) therapy when compared with medical therapy have been summarized^30^ and are re-adapted in *Table 1*. In a total of 21 studies consisting of 1181 patients including two randomized controlled trials,^2,38^ pace-and-ablate therapy with RV apical pacing significantly reduced cardiac symptoms and healthcare use while improving exercise tolerance, QOL, and left ventricular ejection fraction (LVEF).^30^ The success of this strategy stems directly from rhythm regularization, as it is well known that introducing iatrogenic pacing-induced ventricular dyssynchrony in patients with a baseline narrow QRS may simultaneously be harmful, particularly for those with a reduced LVEF. This dyssynchrony may manifest as a reduction in LVEF causing pacing-induced cardiomyopathy (PICM), the prevalence of which is ∼12% increasing to 15%–20% after 5 years in those with normal baseline ventricular function who have >20% pacing.^43–45^ Atrioventricular node ablation and RVP studies therefore may not necessarily account for the development of PICM given the insufficient follow-up duration, the lack of follow-up imaging, and the absence of a widely acknowledged definition of PICM. Nevertheless, AVNA with permanent RVP is currently recommended as a reasonable strategy to control the heart rate in AF irrespective of QRS duration in patients with preserved EF (Class IIa).^46^

**Table 1:** Studies comparing atrial fibrillation node ablation with right ventricle pacing vs. medical therapy (limited to studies including 20 or more patients)

Author, year	No. of pts	Trial design	PAF/permanent AF %/%	EF	Comparison groups	Post-AVNA pacing rate	F/up (m)	Sudden death or early VT/VF, *n* (%)	Total Mort (%)	Conclusions
Geelen^16^ 1997	235	Retr + Pros	0/100	58 ± 16	Analysis of 100 AVNA pts with pacing set at ≤70 vs. prosp. eval of 135 post-AVNA pace set at 90 b.p.m. for 1–3 m (then down to 70 b.p.m. thereafter) with an initial 48 h of in-hospital monitoring		20	6 (6) in post-pacing at ≤70 vs. 0 (0) in 90 b.p.m. group		Sudden death is possible complication of AVNA; appears to be brady-dependent. Malignant arrhythmias can be prevented by temporarily pacing heart at faster HR
Morady^31^ 1993	40	pros RCT	45/55	54 ± 14	Direct current shocks vs. radiofrequency ablation for AVNA	70–120 b.p.m.	12	0 (0)	0 (0)	Radiofrequency ablation more consistent than direct current shocks
Olgin^32^ 1997	54	Retr	-	51 ± 13	RF energy vs. direct current energy for AVNA		24	2 (4)	4 (7)	RF energy as efficacious but safer than direct current energy Sx improvement lower healthcare utilization ↓ ER visits and hospitalizations
Brignole^33^ 1994	23	Pros RCT	0/23	46 ± 11	AVNA vs. medical therapy	VVIR 70–130 b.p.m.	3	0 (0)	1 (4)	Sx improvement and improved exercise capacity
Jensen^4^ 1995	50	Retr	46/54		Retro review of all AVNA	80 b.p.m.	17 (4–36)	2 (4)	6 (12)	Sx improvement and cost effectiveness
Edner^34^ 1995	29	Pros	41/59	45	LVEF and early filling deceleration times (Edec) after 1–2 h of v-pacing at 80 b.p.m. -Pts divided into baseline EF <50% and ≥50%	VVI/VVIR 80 b.p.m. × 1 week and for 1–2 h prior to each echo	7	1 (3)		In those with baseline EF < 50%, EF increased from 32% to 45%. If baseline ≥50% no change in EF
Fitzpatrick^35^ 1996	107	Retr	46/54	51 ± 10	Retro review of all AVNA		2.3 y	3 (3)	17 (16)	Sx and QOL improvement, reduction in hospitalization, reduction in anti-arrhythmics lower EF in pts who died of CV death vs. those who survived long term (35% vs. 51%)
Bubien^36^ 1996	44	Pros	229	?	QOL at 6 mo vs. QOL pre-AVNA		6		2 (4)	Significant sx improvement and QOL
Darpo^37^ 1997	220	Retr	48/52	50 ± 13	Retro review of all AVNA	DDDR or VVIR 70–80 b.p.m. first 1–3 weeks	31	11 (5)	31 (14)	AVNA does not carry a risk of sudden death in pts without structural heart disease
Brignole^38^ 1997	43	RCT	100/0	58 ± 11	DDDR + AVNA vs. drugs	DDDR 70–130	6	0 (0)	0 (0)	Abl + Pm is highly effective and superior to drug therapy for QOL
Buys^39^ 1997	25	Pros	0/100		Exercise capacity pre- and post-AVNA	VVIR	7	0 (0)	0 (0)	↑exercise capacity particularly in those with large baseline LVEDD
Twidale^40^ 1998	22	Pros	0/100	33 ± 9	HF pts with digoxin and ACEi + diuretic and EF < 45%. AVNA vs. AVN mod.		14	2 (9)	2 (23)	AVNA with pacemaker implantation is preferred approach with greater improvement in EF and sx
Lee^2^ 1998	30	RCT	50/50	51 ± 6	AVNA vs. AV node modification without pacemaker	VVIR 70–120	6	0 (0)	0 (0)	EF ↑ both groups, LVESD ↓ QOL↑both groups Sx improve (more so in AVNA pts)
Ablate and Pace Trial Kay^3^ 1998	156	Pros	55/45	50	AVNA on QOL, survival, exercise capacity and vent function	Operator choice	12	5 (3)	23 (15)	Sx improvement with NYHA 2.1–1.9, ↑ QOL, -EF 50 ± 20%–52 ± 20% NS EF 31 ± 20–41 ± 30% no change to exercise duration
Brignole^38^ 1997	66	RCT	100/0		VVIR + AVNA vs. meds	VVIR 70–130 b.p.m.	6	0 (0)	0 (0)	↑QOL with ↓ palpitations, fatigue, dysponea, NYHA
Marshall^41^ 1999	56	RCT	100/0	Fractional shortening 30%	DDDR/MS + AVNA vs. medical therapy	lower rate 70 b.p.m.	4	0 (0)	0 (0)	↑QOL with ↓ palpitations, dysponea. No change in exercise tolerance or EF no change to fractional shortening
AIRCRAFT Weerasooriya^42^ 2003	99	RCT	0/100	54% AVNA 61% med	AVNA vs. medical therapy	VVIR 80–90 b.p.m. × 1 month then as per treating physician	12	3 (3) 2 in AVNA arm, both with low EF; 1 in med arm	3 (3) in total	no change in EF no change in exercise capacity ↑QOL

SD, sudden death; Sx, symptoms; QOL, quality of life; S, significant; NS, non-significant; HF, heart Failure; HFH, heart failure hospitalization; AVNA, atrioventricular nodal ablation; AF, atrial fibrillation; BiV, biventricular pacing; RV, right ventricular pacing; EF, ejection fraction; 6MWD, 6-min walk distance; NYHA, New York Heart Association; Resp, respectively; h, hours; m, months; Retro, retrospective; Pros, prospective; RCT, randomized controlled trial; RF, radiofrequency.

### Atrial fibrillation node ablation and biventricular pacing

Biventricular pacing (BiVP) is considered a superior alternative for patients undergoing AVNA because it effectively addresses the dyssynchrony caused by RVP. Supported by extensive data (*Table 2*), BiVP corrects inter- and intraventricular mechanical dyssynchrony, reduces mitral regurgitation, and contributes to long-term beneficial effects on myocardial remodelling.^55^ The evidence supporting BiVP primarily comes from studies in patients with AV block and reduced LVEF;^56–58^ however, there have been no separate analyses of outcomes for patients with wide vs. narrow baseline QRS. Biventricular pacing in patients with narrow QRS will always result in QRS prolongation that invariably introduces a degree of electrical and likely mechanical dyssynchrony. Harmful effects have been demonstrated when BiVP is performed in patients with a baseline narrow QRS with pre-existing LV dysfunction^59–62^ because slow myocyte-to-myocyte conduction from an LV pacing site will not reproduce normal ventricular activation. Consequently, the 2021 ESC guidelines gives BiVP a Class IIb indication for those undergoing AVNA, *irrespective of QRS duration*, where the LVEF is preserved, a Class IIa for those with mildly reduced LVEF, and a Class I for patients with reduced LVEF.^46^

**Table 2:** Studies comparing biventricular vs. right ventricular pacing for atrioventricular node ablation

Author, year	No. of pts	Trial Design	EF	Pt Population	Experimental group	Control group	1° endpt and pacing rate post	F/up (m)	Conclusions
Leon^47^ 2002	20	Prosp	21 ± 7%	pts with previous AVNA for permanent AF	Upgrade from RV pacing to BiV pacing	none	NYHA, hospitalizations, QOL ECG and echo	17	Mortality 3 (15%) 2 from HF 1 infection ↑QOL; NYHA 3.4–2.4; ↓ HFH EF improvement Mean LVEDD decreased from 683 to 64 (S) and LVESD from 56 to 52 mm.
MUSTIC Linde^48^ 2002	43	RCT	26%	EF < 35% NYHA III, persistent AF AVNA	3-month crossover btw BiV vs. RVp		6MWD	n/a	17-%↑ 6MWD in BiV vs. RVp (S) ↑QOL (S) EF improved by 4%
OPSITE Puggioni^49^ 2004	44	RCT	36.6%	Permanent AF	LVp + AVNA	RVp + AVNA	EF		Regularization improved EF with both LV and RV pacing; modest increase in EF with BiV (BiV: 37%–43% vs. 37%–41% with RVp) Reduction in MR
Simantirakis^50^ 2004	12	Prosp	44%	Permanent AF	LV pacing	RVp + AVNA	Haemodynamics	24 h	Improved LV contractility EF: RVp 40%; LV pacing 49%; BiV pacing 49%
PAVE Doshi^51^ 2005	184	RCT		Permanent AF undergoing AVNA	BiV + AVNA	RVp + AVNA	6MWD	6	BiV significantly improved 6MWD and EF compared with RV Greater benefit with lower EF or symptomatic HF
OPSITE Brignole^52^ 2011	56	Crossover RCT	38 ± 14	Permanent AF undergoing AVNA with or without HF	3-month crossover btw (i) RV and LV pacing (ii) RV and BiV		QOL Exercise capacity	n/a	Rhythm regularization improved QOL and exercise capacity with all modes of pacing LV and BiV pacing provided modest or no additional benefit compared with RV pacing
AVAIL Orlov^53^	153	RCT 4:1 CRT: RV	BiV: 56% RVp 57%	Persistent or permanent AF with AVNA NYHA II–III	BiV pacing	RVp	Echo	6	BiV: EF 56%–59%; RVp: EF 57%–55%. No echo between BiV and RVp ↑LA dilatation in RVp than BiV ↑6MWD in both groups, (NS)
APAF Brignole^54^ 2018	186	RCT	38%	Permanent AF undergoing AVNA with or without HF	BiV pacing	RVp	Composite of Death, HF or HFH	20	1° composite 11% BiV and 26% RVp (S) Similar total mortality ↓HF and HF hospitalizations (S)
APAF-CRT Mortality Trial Brignole^29^ 2021	133	RCT	41%	Permanent AF, narrow QRS, HFH, severe symptoms	BiV pacing	Meds	1° endpt all-cause mortality 2° endpt combined mortality or HFH	29	1° endpt: 11% BiV and 29% drug (CV cause 8% and 17% resp.) 2° endpt: 29% vs. 51% resp.

S, Significant; NS, non-significant; HF, heart failure; HFH, heart failure hospitalization; AVNA, atrioventricular nodal ablation; AF, atrial fibrillation; BiV, biventricular pacing; RV, right ventricular pacing; EF, ejection fraction; 6MWD, 6-min walk distance; NYHA, New York Heart Association; resp, respectively; h, hours; m, months; endpt, endpoint; RVp, right ventricular pacing.

### Atrioventricular node ablation and conduction system pacing

**Figure 2:**
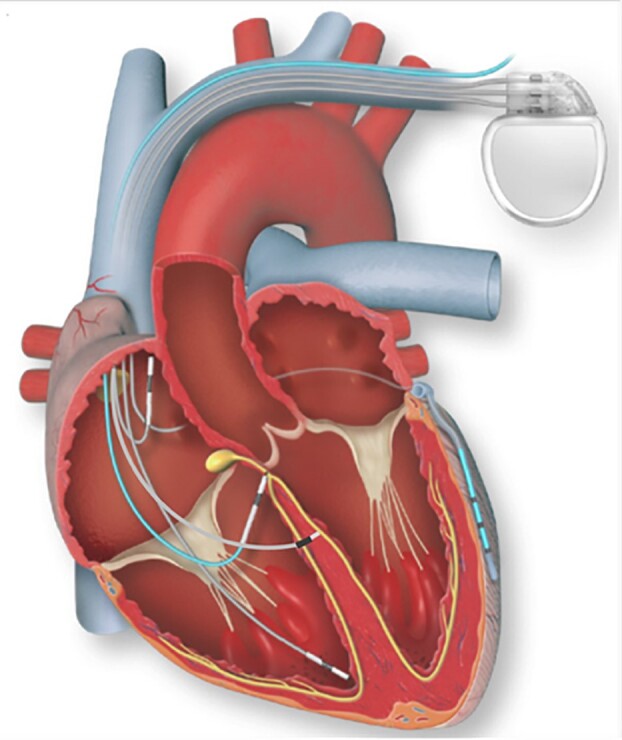
Pacing modalities in patients undergoing atrioventricular node ablation: right ventiricular pacing, his bundle pacing, left bundle branch pacing, and biventricular pacing. See permission letter from Professor Glikson *Figure 2*.^63^

Pacing the proximal conduction system has become a blossoming area of interest (*Figure 2*, *Table 3*). His bundle pacing (HBP) has shown to be a safe and efficacious pacing strategy where direct capture of the His bundle maintains perfect biventricular activation with a resulting narrow QRS. After the initial achievement of HBP in 12 patients with refractory AF undergoing AVNA by Deshmukh *et al*.^64^ HBP has demonstrated in several small randomized trials and larger non-randomized studies to be superior to RVP and even at least as good as BiVP post-AVNA.^78^ Small but significant improvements in LVEF were noted in 38 patients with HBP when compared with BiVP in those with LVEF ≤40% and narrow QRS undergoing a pace-and-ablate strategy in the ALTERNATIVE-AF trial.^73^ Its documented feasibility alongside AVNA however must be considered in the light of the unique challenges posed by the close proximity of the ablation site to the pacing electrode and the general concern for low sensing values with possible atrial oversensing and late rises in capture thresholds frequently necessitating an RV backup lead.^79–81^ The HBP implant remains challenging,^82^ compounded by the limited availability of advanced implantation tools and a decreasing number of specialized operators. Nevertheless, once in experienced hands, success rates for HBP in patients with narrow QRS with AV block are similar to success rates in left bundle branch area pacing (LBBAP).^83^

**Table 3:** Studies to date comparing conduction system pacing with any alternative for atrioventricular node ablation

Author, year	*n*	Trial design	Base EF	Pt population	Exp. group	Cont. group	1° endpt	F/ up (m)	Conclusions
Deshmukh^64^ 2000	14	Prosp	20%	Permanent AF, dilated CM, narrow QRS	HBP + AVNA	n/a	Echo response	23	HBP + AVNA is feasible in pts with dilated CM LVEDD 59–52 mm; LVESD 51–43 mm; EF 20%–31%
Deshmukh^65^ 2004	54	Prosp	23%	Persistent AF, CM, narrow QRS	HBP + AVNA	n/a	Echo response, cardio- pulmonary response	42	↑EF 23%–33% ↓NYHA 3.5–2.2 ↑cardiopulmonary reserve
Occhetta^66^ 2006	18	RCT	52%	AF refractory to medical therapy	6-month crossover between HBP vs. RVp and AVNA		Safety Feasibility 6MWD QOL NYHA	12	No late dislodgements; QRS stable and thresholds 1 ± 0.8 V;0.5 ms vs. 0.68 ± 0.2 V at 12 months ↓interventricular electromechanical delay ↑NYHA 1.7 HBP vs. 2.3 RVp, ↑QOL No change in EF
Occhetta^67^ 2007	68	Retro	51%	AF refractory to medical therapy; narrow QRS	HBP + AVNA	n/a	NYHA, QOL, echo response	21	↑NYHA and QOL No change in EF 1 lead dislodgement interventricular electromechanical delay
Vijayaraman^68^ 2017	42	Retro	45%	AF refractory to medical therapy	HBP + AVNA	n/a	Success Echo response NYHA	19	HBP successful in 95% HBP threshold 1 ± 0.8@1 ms at implant; 1.6 ± 1.2@1 ms at fup EF ↑43–50% NYHA 2.5–1.9
Huang^69^ 2017	42	Prosp	45%	Symptomatic HF with persistent AF	HBP	none	Echo, NYHA, mortality, hosp, HF meds	20	Significant EF improvement EF 56% at 3 months; 60% at 1 year Significant LVEDD reduction Improved NYHA with reduction in diuretic use Reduction in HF hospitalization
Wang^70^ 2019	86	Retro case-control	35%	Persistent AF with HF and ICD indication	HBP + ICD + AVNA	ICD	Safety Echo response, NYHA	30	44 with HBP + ICD; 8 with LBBP + ICD Feasible and safe: 95% success rate ↓LVESV and ↑EF (35%–49%, S) in CSP; ↑EF (39%–43% NS) in ICD arm ↓ HFH, inappropriate shock, death
Su^71^ 2020	81	Prosp	45% ± 14.9	Long-standing persistent AF with HF and narrow QRS	HBP	none	Echo NYHA mortality	Mean 36	Group with reduced LVEF <40%: EF increase from 32% to 49% at 1 yr; 53% last follow-up Group with EF > 40%: EF 57%–62% at 1 year; 65% at last follow-up Improved NYHA class Reduced diuretic and digoxin use 7 pts with HFH and 14 deaths: ↑ risk with lower baseline EF, or higher pulmonary pressures
2021 Senes^72^	24	Prosp propensity match	35% BiV	CRT indication for HF and AVNA	HBP/HBP + LV lead	BiV	Comp of death, HFH, HF	9.6	Composite of death, HFH, or HF 11% in HBP vs. 15% control (NS) No difference in NYHA, QOL and EF (41 vs. 41%).
ALTERNATIVE-AF trial Huang^73^ 2022	50	RCT	32.8 ± 8.9	Persistent AF and LVEF ≤40%, NYHA II–IV, QRS < 120 ms or RBBB	HBP on for 9 mo.	BiV On for 9 mo.	LVEF change NYHA QOL LVEDd BNP	18	↑EF in both BiV and HBP with small statistically sig improvement in EF with HBP, but small number of pts ↑NYHA, QOL, and ↓BNP equivalent for BiV and HBP HBP EF ↑ to 53.9 ± 11.9 at 9 mo. BVP EF ↑ to 51.3 ± 7.4 at 9 mo.
Ivanovski^74^ 2022	50	Retro	39%	Sympt and refractory AF EF < 50% NYHA II–IV QRS < 120 ms	CSP: HBP or LBBP	BiV	QRS Echo NYHA	2–6	12 CRT 28 HBP 10 LBBP Sig narrower QRS with CSP than BiV (105 ms HBP; 127 ms LBBP; 172 ms BiV) No change in NYHA with BiV Significant ↑ NYHA with CSP EF ↑ HBP 39%–49%; ↑LBBP 28%–40%; BiV no change 39%–37%
Vijayaraman^75^ 2022	223	Retro	43 ± 15	Rates refractory to meds, > 6 mo follow-up	CSP	RV		27 ± 19	Pace and ablate performed in the same setting was feasible and safe CSP showed reduction in primary outcome of death or HF hospitalization but RV pacing has a sicker group of patients. CSP: EF 46.5% ± 14–51.9% ± 11 (sig); RV pacing: ↑ 36 ± 16–29% ± 16 (sig)
Qi^76^ 2023	31	Prosp	60%	Persistent AF refractory to 2 ablations; symptomatic	CSP 22 HBP 9 LBBP	none	Symptoms Echo BNP	6	↑ 6MWD scores and EHRA scores No adverse ventricular remodelling Significant improvement in NTproBNP Unchanged EF or LVEDD
Palmisano^77^ 2023	373	Prosp multicentre	41%–42%		CSP: LBBaP (42) or HBP 9 (68)	CRT	incidence of device-related complications Secondary: HF hospitalization, pacing performance, AVNA outcome	12	19 (5.1%) complications (NS): 15 (5.7%) BiV where 8/15 in BiV group related to CS lead; 3 (4.4%) HBP, where 2 of 3 were related to the HBP lead; and 4 (2.4%) in LBBP group with no complications in the LBBP lead. 5.6% AV node conduction recurrence. No difference in battery longevity post-AVNA ↑ BiV EF: 41.7–47.6 ↑HBP EF: 40.8–46.8 ↑LBBaP EF: 42.6–48.4 ↑ Similar in all groups

S, significant; NS, non-significant; HF, heart failure; HFH, heart failure hospitalization; AVNA, atrioventricular nodal ablation; AF, atrial fibrillation; BiV, biventricular pacing; RV, right ventricular pacing; EF, ejection fraction; 6MWD, 6-min walk distance; NYHA, New York Heart Association; resp, respectively; h, hours; m, months; CM, cardiomyopathy; Prosp, prospective; Retro, retrospective; RCT, randomized controlled trial; HBP, His bundle pacing; LBBP, left bundle branch pacing.

Left bundle branch area pacing was introduced after showing it was possible to penetrate the interventricular septum to target the left side of the septum,^84^ and capture the left bundle.^85^ Stimulating the left bundle maintains near-normal LV electrical activation.^86,87^ In the large multicentre MELOS registry, the initial experience from 14 European sites performing LBBAP demonstrated feasibility as a primary technique for both patients with bradycardia and CRT indications where only 1.8% of patients experienced a threshold rise to an absolute value >2.0 V over 18 months of observation.^88^ Vijayaraman *et al.*^75^ demonstrated similar electrical and procedural outcomes of CSP (HBP or LBBAP) vs. conventional pacing (RVP or BiVP) in a retrospective cohort of 223 patients undergoing AVNA. Similar findings were observed in a prospective evaluation where at 12-month follow-up, LBBAP maintained the lowest capture thresholds and longest estimated residual battery longevity with a similar risk of device-related complications and HF hospitalizations.^77^

### Biventricular pacing vs. His bundle pacing vs. left bundle branch area pacing

Despite the level of complexity that BiVP may require pre-AVNA (need for implant expertise and precise tools, suitable coronary sinus branches, and the presence of a complex device with an additional lead), its main default is that it does not avoid the dyssynchrony created in patients with a baseline narrow QRS. While there is a strong rationale for superiority when compared with conventional RVP, when a narrow QRS is obtained, the same cannot be assumed in cases where a non-physiological wide QRS persists post-implant. Furthermore, the baseline QRS in BiV pacing studies was wide (>120 ms), where benefit may have been observed from resynchronization itself. The typical candidate for BiVP is a patient with wide LBBB and HF with reduced EF (HFrEF). In these patients, BiVP is proved to be effective in reducing HF and mortality where electrical uncoupling, septal myocardial scar, or functional conduction block will create lateral activation delays that will never respond to CSP in isolation. In theory, when considering AVNA, BiVP should primarily be used in those with a baseline wide QRS >120 ms and the presence of HF as opposed to *any QRS duration* as suggested in the guidelines and in the absence of data for CSP at the time of writing. Certainly, the reality is more nuanced as CSP does not always achieve a consistently narrow QRS complex particularly in patients with HF with a wide baseline QRS complex. While there are established criteria for successful implantation, particularly for LBBAP, left bundle capture cannot always be obtained.^89^ When suboptimal electrical resynchronization is obtained with CSP due to additional distal conduction delay, septal myocardial scar, or lack of conduction system capture for other reasons, the addition of a lead within the coronary sinus for HBP-optimized CRT (HOT-CRT) or LBBAP-optimized CRT (LOT-CRT) may be necessary.^90,91^ His bundle pacing-optimized CRT resulted in a significant narrowing of the QRS (183 ms at baseline to 162 ms with BiVP, to 151 ms during HBP, to 120 ms with HOT-CRT) with improved LVEF over 14 months of follow-up in patients initially referred for CRT therapy.^70^ Greater resynchronization was similarly seen with LOT-CRT with similar QRS narrowing (182 ms at baseline to 170 ms with BiV and 144 ms with LOT-CRT),^90^ emphasizing the need for further decision aids to attain a more personalized approach to CRT. Further advancements in tools, rigorous training, and implementation of quality control measures will be crucial to ensure that these procedures (BiVP and CSP) are both performed effectively. Finally, until an RCT will prove superiority (or at least non-inferiority) of CSP vs. BiVP, CSP cannot be proposed as a first-line alternative with the intention of CRT for HFrEF either in patients with sinus rhythm or in those with AF.

There also remains ongoing debate about the merits of HBP vs. LBBAP. His bundle pacing results in much higher rates of confirmed conduction system capture and offers pure physiologic activation of both the RV and LV with selective capture in patients with narrow QRS. While left bundle branch pacing (LBBP) ensures physiological activation of the left ventricle, it creates a right bundle activation pattern leading to concern about the lack of physiological activation of the RV. However, recent echocardiographic observations have seen improvements in RV systolic function with LBBAP as evaluated by RV free wall strain.^92^ Propensity score matching of a pace-and-ablate strategy of 99 LBBAP with 86 HBP patients demonstrated similar improvements in echocardiographic and HF outcomes, whereas higher implant success rates, better pacing parameters, and fewer late lead-related complications were present in the LBBAP group.^93^ Importantly, late increased thresholds were noted in 9.3% of the HBP group requiring re-programming to RVP via the previously placed backup lead. Albeit it seems that LBBAP might be more suitable as a first-line CSP strategy for patients undergoing AVNA.^94,95^

## Risks of pace-and-ablate therapy

The pace-and-ablate strategy is often perceived as a harmful therapy as a result of pacemaker dependency, to be avoided at all costs. Apart from the potential for PICM, complications include those related to the device itself: pocket haematoma and infection, pneumothorax, perforation, tricuspid regurgitation, and the need for generator change all in a dependent patient, and AVNA: vascular access complication, lead dislodgement, and rarely sudden cardiac death (SCD). However, the overall complication is low, at 0.8% in the NASPE registry^96^ and 1.8% in the MERFS survey^97^—where these represent historical studies where the incidence of complications over time has likely decreased.

**Figure 3:**
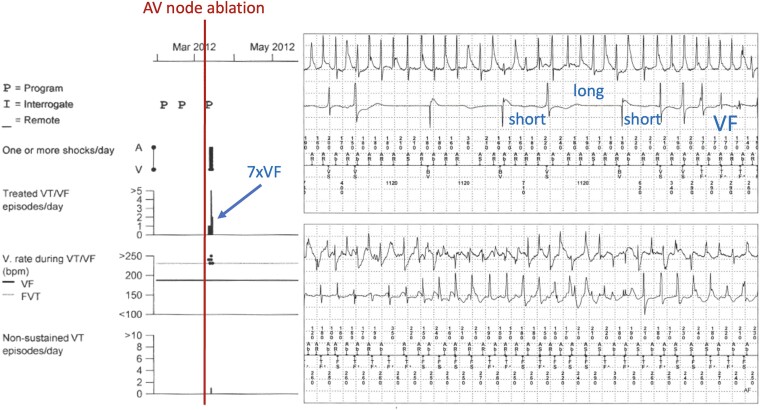
An illustration of a patient who developed polymorphic ventricular fibrillation 1 day after atrioventricular node ablation when the cardiac resynchronization therapy-D device was programmed to a lower rate of 55 b.p.m. post-atrioventricular node ablation. The patient suffered seven ICD shocks in the nights following atrioventricular node ablation

Reports of ventricular arrhythmia and SCD are known, albeit rare, complications of pace-and-ablate therapy (an example of such a case is presented in *Figure 3*). The underlying mechanism most likely contributing to early SCD is that of dispersion of refractoriness due to an abrupt heart rate decrease post-AVNA with abnormally prolonged QT intervals, but underlying cardiac disease or aggravated repolarization abnormalities are certainly contributing causes.^98^ In a large retrospective study evaluating 334 patients with AF who underwent AVNA and pacemaker implantation during the 1990s, nine patients had SCD post-ablation.^99^ Of them, seven were determined to have suffered SCD as a result of the procedure: two within 48 h as out-of-hospital arrests (1.2%) and five in-hospital where two survivors had documented polymorphic ventricular tachycardia (VT) or ventricular fibrillation (VF). In 1997, prevention of post-AVNA malignant arrhythmias was demonstrated by programming devices to a lower rate of 90 b.p.m. for the initial 1–3 months post-AVNA.^16^

## Knowledge gaps and future directions

**Figure 4:**
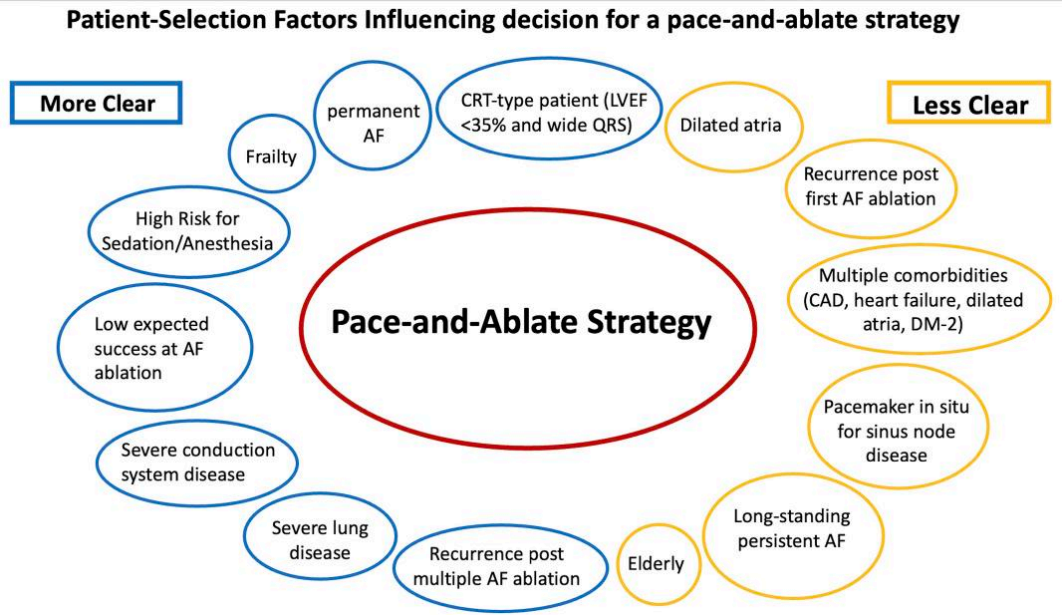
Patient selection factors influencing decision for a pace-and-ablate strategy

The current approach to determine eligibility for pace-and-ablate therapy remains grounded on the chance of AF ablation failure or heightened risk profile as determined by physician experience and opinion, as well as any given centre’s expertise where multiple redo ablation procedures may be part of the normal culture. No formal criteria exist beyond ‘AF ablation non-eligible’. We refer to prior studies (*Tables 1–3*) that consider the safety of the pace-and-ablate approach in addition to the clear benefits of QOL and symptom management. We can similarly consider risk models predicting AF ablation success despite one or more ablations, although their absolute value remains limited to date. When considering a first-line pace-and-ablate approach, it is crucial to account for various patient-specific characteristics (*Figure 4*). Future research on pace-and-ablate therapy should focus on patient selection, timing of the intervention, and long-term follow-up of patient outcomes including stroke and HF hospitalizations, particularly those with less clear indications to proceed with pace-and-ablate therapy.

In patients with HF with reduced EF (HFrEF), CASTLE^25^ and CASTLE-HTX^26^ showed improved clinical outcome after PVI when compared with medical therapy. However, the RAFT-AF^28^ trial failed to observe this benefit. A careful evaluation of these trials reveals different patient profiles and AF types which could explain the differences in outcome. A pace-and-ablate strategy may provide a very reasonable option in select patients with HFrEF.

A number of trials evaluating AVNA + CSP are ongoing. The PACE-FIB trial (Clinicaltrials.gov; NCT05029570) is randomizing 366 patients with HFpEF/HFmrEF and permanent AF to pharmacological rate control or LBBAP with AVNA. The primary outcome measure will be the composite of all-cause mortality, HF hospitalization, and worsening HF at three years.^100^ The ABACUS trial (Clinicaltrials.gov: NCT06207383) will randomize PVI (with additional lesions if deemed necessary) and AVNA + CSP in 220 patients aged >60 years with persistent AF (with at most one previous ablation) and symptomatic HF. The two primary endpoints are superiority of AVNA + CSP for mortality and cardiovascular hospitalizations (including redo procedures) or non-inferiority for mortality and HF hospitalization.

An issue in clinical practice is upgrade to CSP in patients who require AVNA and who are already implanted with a DF-4 implantable cardioverter defibrillator (ICD). These patients may have a device that still has several years of longevity but require a new generator due to the inability to connect the new pacing lead to the existing device. The older DF-1 standard offers this possibility and should be maintained, also for other reasons which are outlined elsewhere.^101^ Direct delivery of LBBAP by ICD leads would avoid the requirement for upgrade. A pilot case series reported successful temporary LBBAP implantation in three of five patients. A lumenless 4.7 F ICD lead is currently being evaluated for RV septal or apical placement.^102^ Due to its resemblance with the 4.2F lumenless 4.2F 3830 lead (Medtronic, MN, USA), this lead may be suitable for LBBAP. However, due to its integrated bipolar design, the entire coil needs to be positioned in the RV to avoid atrial oversensing (which is not an issue with true bipolar ICD leads) and needs to be formally tested for this indication.

Technology is constantly evolving, both in the field of AF ablation and in CSP. The question is to what extent sinus rhythm can be maintained in sick and scarred atria (e.g. atrial cardiomyopathy) and for how long, even with the best of technologies and the most skilled operators. Evolution in lead design and ancillary tools will facilitate CSP implantation (as it did with BiVP) and thereby offer a simple, safe, and pragmatic solution to a growing problem.

## Supplementary data

Supplementary data are not available at *European Heart Journal* online.

## Declarations

### Data Availability

No data were generated or analysed for or in support of this paper.
